# Riding elevators into and out of cells

**DOI:** 10.7554/eLife.62925

**Published:** 2020-10-13

**Authors:** Adam W Duster, Hai Lin

**Affiliations:** 1 Department of Integrative Biology, University of Colorado DenverDenverUnited States; 2 Chemistry Department, University of Colorado DenverDenverUnited States

**Keywords:** membrane transport, membrane protein structure, cryo-EM, x-ray crystallography, *Vibrio cholerae*, *Lactobacillus acidophilus*, *E. coli*

## Abstract

The mechanisms responsible for the trafficking of carboxylate ions across cell membranes are becoming clearer.

**Related research article** Sauer DB, Trebesch N, Marden JJ, Cocco N, Song J, Koide A, Koide S, Tajkhorshid E, Wang DN. 2020. Structural basis for the reaction cycle of DASS dicarboxylate transporters. *eLife* **9**:e61350. doi: 10.7554/eLife.61350

Small carboxylate ions such as citrate and succinate are intermediates in the citric acid cycle, which is a crucial metabolic pathway in aerobic organisms. However, small carboxylate ions also have many other roles: for example, they function as signaling molecules in processes ranging from DNA transcription and replication (Wellen et al., 2009) to heat generation (Mills et al., 2018), and they have also been linked to obesity (Birkenfeld et al., 2011) and seizures (Thevenon et al., 2014).

Cells rely on transmembrane proteins belonging to the DASS family (short for divalent anion sodium symporter) to move small carboxylate ions into and out of cells. There are two clades in the DASS family: cotransporters that import carboxylate ions from the bloodstream into cells (Prakash et al., 2003), and antiporters/exchangers that move some carboxylate ions into cells while moving others out (Pos et al., 1998).

Previously the structure of just one member of the DASS family – a cotransporter called VcINDY, which is found in the bacterium *Vibrio cholerae* – had been determined (Mancusso et al., 2012; Mulligan et al., 2016; Nie et al., 2017). VcINDY contains two subunits, and each of these contains two domains: (i) a scaffold domain, which is anchored in the plasma membrane of the cell and is not, therefore, free to move; (ii) a transport domain, which is more mobile.

It has been predicted that DASS proteins operate with an 'elevator mechanism' that involves the transport domain (to which the carboxylate ion is attached) sliding up and down the scaffold domain between an inward-facing state and an outward-facing state (Figure 1; Mulligan et al., 2016). However, since the structure of VcINDY has only ever been determined for the inward-facing state, evidence in support of this mechanism has remained inconclusive. Now, in eLife, Da-Neng Wang (New York University School of Medicine), Emad Tajkhorshid (University of Illinois at Urbana-Champaign) and co-workers – including David Sauer as first author – report the results of a combined experimental and computational study that helps to shed light on this mystery (Sauer et al., 2020).

**Figure 1.**
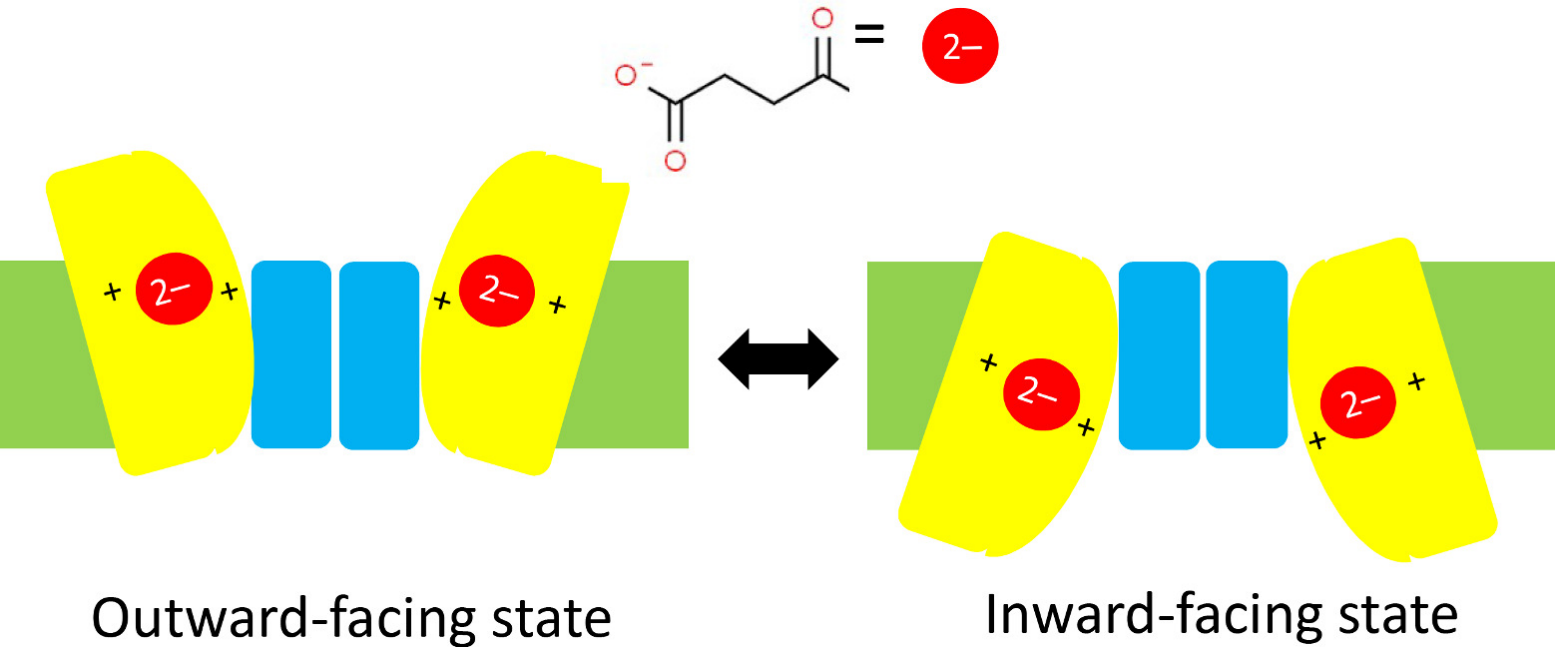
Schematics of the elevator mechanism. Each of the two sub-units in a DASS transporter contains a transport domain (yellow) that is mobile, and a scaffold domain (blue) that is anchored within the plasma membrane of the cell (green). When the transporter is in an outward-facing state (left) two carboxylate ions outside the cell (such as the succinate ion shown here) can each bind to one transport domain and be dragged across the membrane and into the cell by the transport domain as it slides along the scaffold domain. This leaves the transporter is in an inward-facing state (right). In cotransporters the positive charges of two sodium ions in the transport domain have an important role in trafficking carboxylate ions through the membrane; in antiporters/exchangers two positively charged residues have a similar role in the trafficking process. DASS: divalent anion/sodium symporter.

The researchers used a combination of X-ray crystallography and cryo-electron microscopy to determine structures for VcINDY and also for LaINDY, an antiporter that is found in the bacterium *Lactobacillus acidophilus*. Remarkably, they were able to obtain structures for the previously elusive outward-facing state for both. Moreover, they determined the structures when a carboxylate ion was bound to the transport domain and also for the substrate-free case. Relative to the inward-facing state, the transport domain in the outward-facing state is rotated by an angle of 37° and is about 13 Å further away from the inside of the cell (Figure 1).

Sauer et al. then used computer simulations to model the transition from an initial state in which two succinate ions were bound to the outward-facing state in the LaINDY antiporter (based on their experimental structures) to a final state in which the succinate ions were inside the cell and the antiporter was in an inward-facing state: the researchers used an approximate structure for the final state as the actual structure for the inward-facing state in LaINDY has not been determined yet. Jointly, the experiments and simulations lend strong support to the elevator mechanism.

One may ask: how does the cotransporter or antiporter make sure that the carboxylate ion has been loaded into the elevator before the button is pressed? The experimental structures suggest that a 'passport check' is enforced via electrostatic effects. In the absence of the carboxylate ion, the binding site on the transport domain has a positive net charge, which makes it difficult for this domain to pass through the membrane, because charged particles prefer polar environments (such as aqueous solutions) to the nonpolar environments found in the membrane. Loading the negative carboxylate ion onto the binding site neutralizes the postive charge, allowing the transport domain to cruise through. This mechanism limits a form of unproductive transport called slippage (that is, the passage of substrate-free transport domains) in both cotransporters and antiporters.

The work of Sauer et al. also highlights interesting differences between the cotransporter and the antiporter. For the VcINDY cotransporter the positive charge on the transport domain is provided by sodium ions, and the binding of the carboxylate ion to the transport domain leads to large changes in conformation. It also appears that the relatively weak initial binding of sodium ions in the cotransporter is strengthened by the arrival of the carboxylate ion. In contrast, the positive charges in the LaINDY antiporter are provided by amino acid residues rather than sodium ions, and the conformational changes caused by bindin﻿g appeared to be small and local.

It would be interesting to explore the effects of introducing mutations to the residues at the sites of the sodium ions in VcINDY cotransporters or the equivalent sites in LaINDY antiporters. Would it be possible to engineer a sodium-independent cotransporter? And could a cotransporter be converted into an antiporter, and vice versa? Answers to these questions will lead to a deeper understanding of the structure-function relationships for proteins belonging to the DASS family.
